# Clotting, inflammation, and immunity: the multifaceted role of platelets in HIV and sickle cell disease – a narrative review

**DOI:** 10.1097/MS9.0000000000003677

**Published:** 2025-08-04

**Authors:** Emmanuel Ifeanyi Obeagu

**Affiliations:** Department of Biomedical and Laboratory Science, Africa University, Zimbabwe

**Keywords:** HIV, immunity, inflammation, platelets, sickle cell disease

## Abstract

Platelets, long recognized for their function in hemostasis, are increasingly acknowledged for their role in immune modulation and inflammation, especially regarding complex co-infections. This review examines the complex role of platelets in co-infection with HIV and sickle cell disease (SCD), highlighting their functions that go beyond coagulation and contribute to chronic inflammation, immune activation, and vascular dysfunction. In HIV, there is an increase in platelet activation, fostering inflammation and endothelial dysfunction, thereby raising the likelihood of thrombotic events. Likewise, in SCD, platelets play a role in vaso-occlusion, which worsens the microvascular issues associated with the disease. The overlap of HIV and SCD complicates platelet function, enhancing both pro-coagulant and pro-inflammatory pathways. In SCD, platelet aggregation speeds up endothelial damage, and immune activation from HIV worsens this condition, increasing the chances of thromboembolic incidents. Platelets engage with immune cells, enhancing T cell activation and HIV viral replication, while creating an inflammatory environment that exacerbates the pathology of both conditions. These interactions establish a detrimental cycle of platelet dysfunction, inflammation, and tissue injury, which presents considerable difficulties for patient care.

## Introduction

Platelets have been acknowledged for their crucial function in hemostasis and thrombosis for a long time. Historically regarded as anucleate remnants stemming from megakaryocytes, their main role was perceived to be the preservation of vascular integrity via clot formation. Nevertheless, an increasing amount of evidence accumulated over the last twenty years has considerably broadened this perspective, establishing platelets as active participants in immune monitoring, inflammation, and defense against pathogens. Their engagement with immune cells, cytokines, and the endothelium indicates that these small cellular fragments have an unexpectedly significant impact on the pathophysiology of numerous chronic and infectious illnesses^[1,2]^. HIV infection and sickle cell disease (SCD) are among the conditions where platelet activation and dysfunction are critical. HIV, a long-lasting viral infection, is recognized for its ability to provoke ongoing immune activation and inflammation, even with viral suppression from antiretroviral treatment. Likewise, SCD is marked by repeated occurrences of vaso-occlusion, hemolysis, and ongoing inflammation, all of which engage platelet activation and interactions with the coagulation cascade. Although they have different causes, both diseases exhibit similar pathways that involve endothelial damage, cytokine production, and thromboinflammation, placing platelets at the core of these interconnected processes^[3,4]^.

**HIGHLIGHTS**

- Platelet activation in disease – platelets contribute to hypercoagulability in HIV and vaso-occlusive crises in SCD, worsening disease severity.
- Inflammatory mediators – platelets release cytokines and promote immune cell activation, driving chronic inflammation.
- Endothelial dysfunction – platelet-derived microparticles enhance vascular injury in both HIV and SCD.
- Immune modulation – platelets interact with immune cells, influencing HIV persistence and immune exhaustion in SCD.
- Therapeutic potential – anti-platelet and immunomodulatory therapies offer promising strategies for managing platelet-related complications.

In HIV, platelets are activated and show modified transcriptomic and proteomic characteristics. They release higher amounts of pro-inflammatory mediators like CD40L and IL-1β and create clusters with leukocytes that lead to immune dysfunction and vascular damage. Moreover, HIV-related thrombocytopenia and elevated platelet turnover are not just consequences of infection but play active roles in the advancement of the disease. In HIV, platelet-derived microparticles (PMPs) have been associated with tissue injury and immune regulation, emphasizing the idea that platelets play a crucial role in the development of HIV-related comorbid conditions^[5,6]^. Likewise, in SCD, activation of platelets is a regular observation, especially during vaso-occlusive crises. Hemolysis results in the liberation of free heme along with other damage-associated molecular patterns (DAMPs), which subsequently stimulate toll-like receptors (TLRs) and additional pro-inflammatory pathways in platelets. Activated platelets emit thromboxane A2, serotonin, and other substances that intensify vasoconstriction and encourage interactions with neutrophils and monocytes. These interactions between platelets and leukocytes lead to endothelial dysfunction, hypercoagulability, and chronic inflammation, all of which are characteristic features of SCD pathophysiology^[7,8]^.The scenario grows more complicated in individuals with both HIV and SCD coinfection, as the combined inflammatory, thrombotic, and immunologic disruptions may work together to deteriorate clinical results. Though research on platelet biology in this dual-disease scenario is still scarce, existing evidence indicates that the combined effects of inflammation from viral infection and hemolysis exacerbate platelet dysfunction. Grasping how platelets influence immune and vascular responses in co-infected individuals is crucial for developing targeted treatments and minimizing disease impact^[9,10]^.

## Aim

The aim of this review is to explore the multifaceted role of platelets in immune system modulation, with a focus on their interactions with immune cells, cytokine release, and influence on both the innate and adaptive immune responses.

## Review methods

This narrative review was conducted through a systematic approach, gathering relevant studies, experimental data, and clinical reports to examine the role of platelets in immune system modulation and their implications in various disease conditions, including HIV, SCD, and chronic inflammatory disorders.

## Literature search strategy

A comprehensive literature search was performed using online databases including PubMed, Google Scholar, Scopus, and Web of Science. Keywords used in the search included “platelets,” “immune modulation,” “inflammation,” “cytokine release,” “HIV,” “Sickle Cell Disease,” “autoimmune disorders,” and “immune activation.” Both human and animal studies were considered, with a preference for studies focusing on platelet function in immune responses and disease pathogenesis.

## Inclusion and exclusion criteria

Inclusion criteria for studies were as follows:

1. Original research articles, reviews, and meta-analyses focused on the role of platelets in immune modulation and inflammation.
2. Studies that explored the interaction of platelets with immune cells (e.g., leukocytes, monocytes, T cells) and their impact on disease progression in conditions such as HIV, SCD, and autoimmune disorders.
3. Research reporting the mechanisms of platelet involvement in immune responses, cytokine release, and the regulation of inflammation.

Exclusion criteria:

1. Studies focused on non-human subjects, unless relevant to human disease models.
2. Studies that did not focus on platelets or immune modulation in the context of inflammatory diseases.
3. Articles published prior to 2000.

## Platelets and coagulation in HIV and sickle cell disease

Platelets are critical for normal hemostasis, playing a key role in the formation of blood clots to stop bleeding. However, in the context of chronic diseases such as HIV and SCD, platelet function becomes dysregulated, contributing to a range of complications beyond their traditional role in coagulation. Both HIV and SCD induce changes in platelet activation and function, which significantly affect the coagulation pathways and increase the risk of thromboembolic events. The interplay between platelets and coagulation in these two diseases, particularly when co-existing, is a complex and evolving area of research[11]. In HIV, the viral infection leads to chronic immune activation, which directly affects platelet function[12]. Platelets in HIV-positive individuals often exhibit increased activation, which is associated with higher levels of platelet-derived microparticles and an enhanced propensity for thrombosis. HIV-induced platelet activation contributes to vascular damage and endothelial dysfunction, promoting thrombus formation and raising the risk of cardiovascular events.

In addition to these effects on coagulation, platelets in HIV-infected individuals interact with immune cells, contributing to the chronic inflammation seen in these patients. This immune activation further exacerbates the imbalance between procoagulant and anticoagulant pathways, leading to a heightened risk of clot formation despite the presence of an ongoing inflammatory state[13]. Sickle cell anemia leads to the chronic formation of sickle-shaped red blood cells, which are rigid and prone to aggregation. This aggregation causes microvascular occlusion, and platelets are crucial players in this pathological process. They contribute to the formation of thrombi in small blood vessels, which can lead to organ damage and vaso-occlusive crises, one of the hallmark complications of SCD. Platelet activation in SCD is also closely tied to the inflammatory environment in these patients. Inflammatory mediators such as cytokines and leukocytes interact with platelets, exacerbating their activation and contributing to endothelial injury. This process amplifies the risk of thrombosis, even in the absence of traditional risk factors for clotting[14].

The combination of HIV and SCD creates a unique challenge in platelet function and coagulation. When both diseases co-exist, the dysregulation of platelet activation is amplified. HIV infection exacerbates the inflammatory environment in SCD, further heightening platelet activation and the propensity for thrombus formation. The co-occurrence of these two diseases increases the likelihood of thromboembolic events, such as stroke or deep vein thrombosis, which are already more common in SCD patients. Moreover, platelet dysfunction in this dual context may also compromise immune responses and contribute to an increased risk of infections, a common complication in both HIV and SCD[15]. The coagulation system in individuals with HIV and SCD is characterized by an imbalance between procoagulant and anticoagulant factors. In HIV-infected individuals, the chronic immune activation seen in the disease promotes the generation of procoagulant factors, while simultaneously impairing the function of natural anticoagulants such as protein C and antithrombin. This results in a hypercoagulable state despite the presence of inflammation. In SCD, a similar phenomenon occurs, where platelet activation and aggregation occur alongside a depletion of anticoagulant proteins, creating an environment conducive to thrombus formation. Additionally, the high levels of fibrinogen and other acute-phase reactants in both HIV and SCD further promote the risk of clot formation[16].

## Platelet inflammation and immune activation in HIV

Platelets, once primarily associated with their role in blood clotting and hemostasis, have recently been recognized as key players in inflammation and immune activation, particularly in chronic infections such as HIV. The relationship between platelets, inflammation, and immune activation in HIV is complex, as platelets not only participate in clot formation but also act as mediators of inflammatory responses that contribute to the pathophysiology of the disease. In HIV-infected individuals, platelets become hyperactivated, which exacerbates both the inflammatory state and the immune dysfunction characteristic of the infection[17]. Platelets in HIV-positive individuals exhibit increased activation, which manifests as elevated platelet aggregation and the release of inflammatory mediators such as cytokines, chemokines, and platelet-derived microparticles. These microparticles play an important role in immune cell recruitment and activation, facilitating chronic inflammation and immune dysregulation. The enhanced activation of platelets also promotes the interaction between platelets and leukocytes, particularly monocytes and T lymphocytes. This interaction is critical for the persistence of immune activation and viral replication. Platelet-leukocyte aggregates have been shown to amplify the inflammatory environment, contributing to the elevated levels of pro-inflammatory markers often observed in HIV-infected patients, even during antiretroviral therapy (ART)[18].

Beyond their direct effects on inflammation, platelets also influence immune activation by engaging with the endothelial cells that line blood vessels. In HIV, this interaction can lead to endothelial dysfunction, which facilitates the formation of a prothrombotic state and increases the risk of cardiovascular events, a known complication of HIV infection. Platelet activation also enhances the expression of adhesion molecules, which further promotes the recruitment and activation of immune cells to sites of infection or inflammation. As a result, platelets act as both effectors and amplifiers of immune responses, contributing to the chronic low-grade inflammation and immune activation observed in HIV-positive individuals, even when viral replication is well controlled[19]. Moreover, platelets in HIV infection also have a direct effect on viral persistence. Through interactions with immune cells, platelets facilitate the retention of HIV in lymphoid tissues, thereby contributing to the establishment of viral reservoirs. Platelet activation in these tissues is thought to assist in viral replication by promoting a local inflammatory microenvironment that supports the persistence of HIV. The chronic immune activation and inflammation associated with this process not only impairs immune system function but also enhances the risk of opportunistic infections and malignancies, common concerns in HIV-infected patients^[20,21]^.

Platelet-driven inflammation in HIV infection is also associated with several comorbidities, including cardiovascular disease, neurocognitive disorders, and renal dysfunction. Platelet activation exacerbates endothelial injury and promotes atherosclerotic plaque formation, leading to increased cardiovascular risk. In the brain, platelet activation contributes to neuroinflammation, which is linked to cognitive decline and other neurological complications observed in HIV-positive individuals. Additionally, platelets contribute to the progression of HIV-associated nephropathy, in which platelet aggregation in the glomeruli and tubules accelerates kidney damage[22]. Therapeutically, the modulation of platelet activation and inflammation presents a promising avenue for managing HIV-related complications. Although antiplatelet agents such as aspirin have been used to reduce thrombotic events in HIV-positive patients, their use requires careful consideration due to the increased risk of bleeding in this population. The potential for targeting platelet-mediated inflammation through novel therapies – such as platelet inhibitors or anti-inflammatory cytokine treatments – may provide new opportunities for preventing cardiovascular, renal, and neurological complications in HIV-infected individuals.

## Platelet contributions to vascular injury in sickle cell disease

While the primary pathological mechanism of SCD is related to the abnormal hemoglobin S and the resulting deformation of red blood cells, platelets play a crucial secondary role in amplifying vascular injury. Platelet activation and aggregation are central to the development of thrombotic events and vascular damage in SCD, significantly contributing to the morbidity associated with the disease[23]. Platelets in SCD are often hyperactivated, which is a key factor in the pathogenesis of vascular injury. This activation is driven by several factors, including the presence of sickle cells in circulation, the inflammatory environment, and endothelial dysfunction. In SCD, the blood vessels are frequently subjected to shear stress due to the rigid, sickle-shaped red blood cells. This stress causes endothelial damage, exposing subendothelial collagen and promoting platelet adhesion and activation. Platelets adhere to the injured endothelium and become activated, releasing pro-inflammatory mediators, such as thromboxane A2, which amplify the local inflammatory response. The result is a vicious cycle of platelet aggregation and endothelial injury, further promoting vascular occlusion and contributing to the development of painful vaso-occlusive crises, which are characteristic of SCD[24]. The interaction between platelets and sickle cells exacerbates thrombus formation and contributes to microvascular occlusion in SCD. Platelets interact with sickle-shaped red blood cells by binding to the exposed phosphatidylserine on the surface of these cells, a marker of cell activation. This interaction leads to the formation of platelet-rich thrombi in small blood vessels, resulting in obstruction of blood flow and tissue ischemia. The thrombi formed in these microvessels can obstruct blood flow, causing further tissue damage and contributing to the recurrent pain crises observed in SCD patients. Platelets also contribute to the propagation of clot formation by promoting the activation of the coagulation cascade, leading to the formation of fibrin clots and increasing the risk of thrombosis[25].

In addition to their role in thrombus formation, platelets in SCD also contribute to the inflammatory response that underlies the disease’s vascular complications. Platelet-derived microparticles, small vesicles released upon platelet activation, have been shown to carry inflammatory mediators that can activate leukocytes and amplify the immune response. These microparticles can bind to endothelial cells, further contributing to endothelial activation and dysfunction. As a result, platelets in SCD are not merely passive participants in clot formation but active contributors to the inflammatory milieu that exacerbates vascular injury. The chronic inflammatory state in SCD leads to increased platelet activation, perpetuating a cycle of vascular damage that extends beyond the acute vaso-occlusive events[26]. Furthermore, platelets in SCD contribute to the development of endothelial dysfunction. The activation of platelets in the presence of sickle cells leads to the release of pro-inflammatory cytokines and growth factors, such as platelet-derived growth factor (PDGF) and transforming growth factor-beta (TGF-β). These mediators promote smooth muscle cell proliferation and collagen deposition, processes that contribute to intimal thickening and vascular remodeling. Over time, this vascular remodeling can result in chronic damage to blood vessels, contributing to long-term complications such as stroke, organ damage, and pulmonary hypertension in SCD patients. Platelets, therefore, play a key role not only in the acute events of vaso-occlusion but also in the chronic vascular damage that underpins the progressive nature of the disease[27].

Therapeutically, targeting platelet activation has emerged as a potential strategy for reducing vascular injury in SCD. Anti-platelet agents such as aspirin have been explored in clinical trials as a means to reduce the risk of thrombotic complications in SCD, with some studies showing a reduction in pain crises and stroke risk. However, the use of anti-platelet therapy in SCD is complex, as platelets are essential for normal hemostasis and excessive inhibition of platelet function could lead to bleeding complications. Therefore, therapies that specifically target platelet activation without broadly inhibiting platelet function are of particular interest. These approaches could help reduce platelet-induced vascular injury while minimizing the risk of bleeding[28]. In addition to anti-platelet therapies, other potential interventions include agents that modulate the inflammatory environment in SCD. For instance, targeting the inflammatory pathways that activate platelets, such as the cytokine interleukin-1 (IL-1) or the complement system, may help reduce platelet activation and subsequent vascular injury. Furthermore, the development of therapies aimed at preventing platelet-sickle cell interactions could be another promising avenue for reducing thrombus formation and improving blood flow in SCD patients.

## Interactions between platelets, HIV, and sickle cell disease

Platelets, traditionally known for their role in hemostasis, are emerging as key mediators of inflammation and immune modulation in various diseases. In both HIV and SCD, platelets are implicated in enhancing the pathological processes associated with these disorders. The interactions between platelets, HIV, and SCD represent a complex interplay that exacerbates both the inflammatory environment and the thrombotic tendencies associated with each condition. The convergence of platelet activity in the context of HIV and SCD highlights the multifactorial role of platelets not only in clotting but also in immune activation, inflammation, and tissue damage[29]. In the case of HIV, platelets undergo hyperactivation, which contributes to a pro-inflammatory state that is exacerbated by viral replication and immune dysregulation. The elevated levels of activated platelets release various mediators such as cytokines, chemokines, and microparticles, which propagate immune activation and contribute to the chronic inflammation seen in HIV. This hyperactivation is compounded by the endothelial dysfunction induced by HIV, where the blood vessels become more permeable, allowing for platelet adhesion and further activation. In SCD, the pathophysiology is similarly driven by inflammatory and thrombotic processes. The presence of sickle-shaped red blood cells, which cause microvascular occlusion and endothelial damage, also stimulates platelet activation and aggregation. Platelets, in turn, amplify the vascular injury by releasing pro-inflammatory mediators and interacting with leukocytes and sickle red blood cells[30].

When HIV and SCD co-occur, their effects on platelet function are compounded. In individuals with both conditions, the presence of HIV can enhance platelet activation and further promote the inflammatory state, while SCD provides a hypercoagulable environment with endothelial dysfunction. Platelets in co-infected individuals may show heightened activation, contributing to an even more severe inflammatory environment. For example, platelets in HIV/SCD co-infection have been shown to interact with activated T cells and monocytes, facilitating the persistence of chronic inflammation. The release of platelet-derived microparticles in such patients further exacerbates this inflammation by promoting additional platelet activation, leukocyte recruitment, and endothelial injury. This vicious cycle of platelet-driven inflammation can lead to worsened complications, including increased risk of thrombotic events, vascular damage, and tissue ischemia in both HIV and SCD[31]. Moreover, platelets play a role in facilitating HIV viral persistence and replication in tissues. In HIV-infected individuals, platelets interact with infected immune cells, enhancing viral replication and contributing to the formation of viral reservoirs in lymphoid tissues. This process is amplified in individuals with SCD, where the heightened platelet activation and the pro-thrombotic environment facilitate the retention of infected cells and viral particles. These interactions further complicate the immune response and hinder efforts to fully suppress viral replication. Additionally, the chronic immune activation in both HIV and SCD promotes endothelial dysfunction, a hallmark of both conditions, with platelets exacerbating this dysfunction by releasing growth factors and inflammatory mediators that promote vascular remodeling and damage[32].

In SCD, the hyperactivation of platelets also contributes to the formation of platelet-rich thrombi, which can obstruct blood flow in small vessels, leading to painful vaso-occlusive crises. When compounded by the effects of HIV, the risks of microvascular occlusion, thrombosis, and tissue ischemia become even more pronounced. Platelet-leukocyte aggregates are more prevalent in individuals with HIV and SCD co-infection, which promotes not only thrombotic events but also sustained inflammation and tissue damage. In this context, platelets become not just passive agents in clot formation but active participants in the chronic inflammatory milieu, promoting endothelial dysfunction, microthrombosis, and ischemic injury[33]. Therapeutically, managing platelet activity in the context of HIV and SCD co-infection is challenging. Anti-platelet therapy, such as the use of aspirin or clopidogrel, has been considered for reducing thrombotic events in both HIV and SCD patients. However, the use of these agents requires careful monitoring, as inhibiting platelet function can increase the risk of bleeding, particularly in individuals who may have underlying vascular or organ dysfunction due to either HIV or SCD. Newer therapies targeting specific platelet activation pathways, such as inhibitors of platelet receptors involved in platelet aggregation or the release of inflammatory mediators, may provide more targeted interventions for reducing platelet-driven inflammation and thrombosis. These strategies hold promise for reducing complications in co-infected patients by modulating platelet function without compromising normal hemostasis[33].

## Specific molecular mechanisms: CD40L, TLR4, IL-1β, and P-selectin

Grasping the molecular foundations of platelet activity in HIV and SCD necessitates a detailed analysis of particular mediators that connect thrombosis, inflammation, and immune regulation. CD40 ligand (CD40L), Toll-like receptor 4 (TLR4), interleukin-1 beta (IL-1β), and P-selectin have been identified as key components in the pathophysiology driven by platelets^[34]^.

### CD40 Ligand (CD40L)

CD40L, or CD154, is a transmembrane protein found on activated platelets and is cleaved into a soluble form (sCD40L), with both having strong immunomodulatory effects. In both HIV and SCD, increased levels of sCD40L have been noted, leading to systemic inflammation and activation of the endothelium. CD40L binds to CD40 on monocytes, dendritic cells, and endothelial cells, stimulating the secretion of pro-inflammatory cytokines, expression of tissue factor, and enhancement of adhesion molecules. In HIV, elevated sCD40L correlates with immune activation and sustained viral presence, whereas in SCD, it worsens endothelial dysfunction and vaso-occlusion. The CD40/CD40L pathway serves as a central junction for thrombo-inflammatory signaling in both conditions^[35,36]^.

### Toll-Like Receptor 4 (TLR4)

Platelets exhibit functional TLR4, a pattern recognition receptor that detects lipopolysaccharide (LPS) and endogenous DAMPs, such as heme and high-mobility group box 1 (HMGB1). In SCD, exposure to heme from hemolysis can trigger platelet TLR4, resulting in NF-κB activation and the release of pro-inflammatory cytokines as well as the formation of platelet-leukocyte aggregates. In HIV, chronic immune stimulation and microbial translocation may similarly activate TLR4 on platelets, leading to persistent inflammation. The activation of TLR4 increases platelet reactivity and prepares them to engage with leukocytes, a mechanism that drives the immunopathogenesis of both diseases^[37,38]^.

### Interleukin-1 Beta (IL-1β)

IL-1β is a key pro-inflammatory cytokine secreted after inflammasome activation. Platelets can express and secrete IL-1β, either directly or through the transfer of microparticles to immune cells. In both HIV and SCD, IL-1β from platelets plays a role in activating endothelial cells, attracting monocytes, and enhancing vascular inflammation. Significantly, the NLRP3 inflammasome, responsible for IL-1β maturation, gets activated in both conditions due to oxidative stress, hypoxia, and stimuli associated with pathogens. The release of IL-1β driven by platelets serves as a crucial mediator that connects innate immunity to thromboinflammation in HIV and SCD^[39,40]^.

### P-selectin (CD62P)

P-selectin is kept in platelet α-granules and quickly moved to the platelet surface when activated. It facilitates the attachment of platelets to leukocytes through P-selectin glycoprotein ligand-1 (PSGL-1), promoting the development of platelet-leukocyte aggregates. Increased levels of surface P-selectin have been noted in individuals with HIV and patients with SCD, and this correlates with heightened vascular inflammation, monocyte activation, and damage to the endothelium. Additionally, P-selectin interaction promotes leukocyte rolling and migration, aiding the infiltration of inflammatory cells into tissues. In SCD, P-selectin is crucial in vaso-occlusive events and has emerged as a target for therapeutic inhibition (e.g., crizanlizumab). Its dual function in hemostasis and immune modulation renders it a crucial molecular switch in platelet-related pathology^[41,42]^.

## Implications for therapeutic strategies

As both HIV and SCD are characterized by inflammatory and thrombotic tendencies, therapeutic strategies must address both the underlying viral infection and the hematologic complications associated with SCD. Platelets, as central mediators of inflammation, immune activation, and thrombosis in both conditions, represent a promising therapeutic target. However, given the multifaceted role of platelets in these diseases, developing effective strategies to manage their activity without compromising hemostasis or exacerbating other complications presents a significant challenge[34]. One potential therapeutic approach is the use of anti-platelet agents, which could help mitigate platelet-driven inflammation and thrombus formation in HIV/SCD co-infected patients. Aspirin, a well-known anti-platelet agent, has been used in various thrombotic conditions, including SCD, to reduce the incidence of painful crises and vascular events. While aspirin may offer benefit by decreasing platelet aggregation and reducing inflammation, its use in HIV/SCD patients must be approached with caution due to potential side effects, including an increased risk of bleeding. Moreover, HIV itself can lead to gastrointestinal complications, which may increase the risk of bleeding in patients treated with aspirin or other nonsteroidal anti-inflammatory drugs (NSAIDs). Therefore, the clinical decision to use anti-platelet therapy must balance the risk of thromboembolic events with the potential for bleeding, requiring careful monitoring[35].

Beyond traditional anti-platelet therapies, newer approaches are exploring targeted interventions to modulate specific pathways involved in platelet activation. For instance, platelet GPIIb/IIIa inhibitors, which prevent platelet aggregation, have been explored in conditions with excessive platelet activation. These inhibitors have shown promise in reducing thrombotic events in other diseases, but their safety and efficacy in HIV/SCD co-infected patients remain uncertain. Another promising strategy involves targeting the pro-inflammatory mediators released by activated platelets, such as thromboxane A2, platelet-derived growth factor (PDGF), and cytokines. Inhibiting these mediators could potentially reduce both platelet activation and the downstream inflammatory response, addressing the chronic inflammation seen in both HIV and SCD. Further studies are needed to assess the feasibility and safety of these agents in patients with HIV and SCD, particularly in terms of their ability to reduce vascular injury and improve overall disease management[36]. In addition to pharmacologic approaches targeting platelets, there is growing interest in managing the underlying inflammatory and immune dysregulation that drives platelet activation in both HIV and SCD. Anti-inflammatory therapies, such as corticosteroids or biologics targeting specific cytokines (e.g., TNF-α or IL-1), could be beneficial in reducing the inflammatory environment that promotes platelet hyperactivation. For instance, in HIV, immune activation is a significant contributor to platelet activation, and therapies that reduce the chronic inflammation associated with HIV infection may help normalize platelet function. Similarly, in SCD, managing the chronic inflammatory state through therapies that reduce the production of pro-inflammatory cytokines or inhibit inflammatory cell recruitment could lessen platelet activation and reduce the incidence of vaso-occlusive crises. However, the use of such therapies must be carefully tailored to avoid adverse effects, such as immune suppression or exacerbation of infections, particularly in HIV-infected patients[37].

Moreover, strategies aimed at improving endothelial function may complement platelet-targeted therapies in both HIV and SCD. In both diseases, endothelial dysfunction contributes to platelet activation and the formation of thrombi. Interventions aimed at restoring endothelial integrity, such as endothelial cell-protective agents, could reduce the interactions between platelets and the vessel wall, thereby limiting thrombotic events. Medications that promote nitric oxide production or inhibit endothelial cell apoptosis have shown promise in preclinical models of vascular disease, and similar approaches may be beneficial in HIV/SCD co-infected patients[38]. The possibility of combining therapies to address both platelet activation and the underlying disease processes presents another therapeutic avenue. For example, ART is a cornerstone of HIV management, and its role in reducing immune activation and inflammation could help mitigate platelet activation. In SCD, hydroxyurea is used to reduce the frequency of vaso-occlusive crises and improve hematologic parameters, and its potential to influence platelet function warrants further investigation. Combining ART with hydroxyurea or other disease-modifying therapies may offer synergistic benefits in reducing platelet-driven pathology in co-infected individuals. Furthermore, the incorporation of personalized medicine approaches, guided by biomarkers of platelet activation and inflammation, could optimize treatment regimens and improve patient outcomes[38].

## Platelet role in immune system modulation

Platelets, traditionally recognized for their role in hemostasis and thrombus formation, are now increasingly recognized for their complex role in immune system modulation. In addition to their participation in blood clotting, platelets have emerged as key players in immune responses through their interactions with immune cells, cytokine release, and modulation of inflammatory processes. Platelets are able to communicate with various immune cells, including leukocytes, monocytes, and dendritic cells, facilitating both the innate and adaptive immune responses. This immunomodulatory function is essential in maintaining homeostasis during normal immune surveillance but can also contribute to pathological conditions when dysregulated[39]. One of the primary mechanisms by which platelets influence immune responses is through the release of cytokines, chemokines, and growth factors stored in their granules. When activated, platelets release a variety of bioactive molecules, including platelet-derived growth factor (PDGF), transforming growth factor-beta (TGF-β), interleukins (IL-1, IL-6), and tumor necrosis factor-alpha (TNF-α). These molecules influence immune cell activation and recruitment to sites of infection or injury. Platelets can also enhance the recruitment and activation of neutrophils, monocytes, and T cells by secreting specific chemokines such as IL-8 and RANTES. This ability to influence immune cell function allows platelets to play a critical role in the inflammatory response, not only in response to acute injury but also in chronic inflammatory diseases, including autoimmune disorders and infections[40].

Platelets also interact with antigen-presenting cells (APCs) such as dendritic cells, facilitating the initiation and regulation of adaptive immune responses. Upon activation, platelets can express surface receptors such as CD40, CD62P (P-selectin), and CD154 (CD40 ligand), which are involved in interactions with dendritic cells and other immune cells. These interactions can influence the differentiation of T cells, including the balance between T helper (Th)1 and Th2 responses, which are critical in both protective immunity and the pathogenesis of inflammatory diseases. Additionally, platelets have been shown to interact with B cells, modulating antibody production and influencing immune memory. These interactions suggest that platelets play an active role in the adaptive immune response, regulating the magnitude and duration of immune reactions[41].

In addition to their role in promoting immune responses, platelets are also involved in the resolution of inflammation. By releasing anti-inflammatory cytokines such as TGF-β and IL-10, platelets can help modulate immune cell activity and dampen excessive inflammation. This dual role of promoting and resolving inflammation highlights the complexity of platelet function in the immune system. Dysregulated platelet activation, however, can lead to pathological immune responses, such as in autoimmune diseases, chronic inflammation, and in infections like HIV or sepsis. In these conditions, platelet hyperactivation can contribute to tissue damage, prolonged inflammation, and the development of immune-mediated pathologies[42]. Furthermore, platelets are involved in the formation of immune complexes and the clearance of apoptotic cells, both of which are important for maintaining immune tolerance and preventing autoimmunity. In autoimmune diseases such as systemic lupus erythematosus (SLE), platelets can bind to autoantibodies, forming immune complexes that can exacerbate tissue damage.

In healthy individuals, however, platelets help clear these complexes and apoptotic cells from the bloodstream, preventing the activation of autoreactive immune cells and the development of autoimmune responses. This clearance mechanism is essential for the maintenance of immune tolerance and the prevention of immune dysregulation[43]. The role of platelets in immune modulation extends to various infections, including viral infections like HIV. In HIV, platelets are not only involved in coagulation but also in immune activation. Activated platelets in HIV-infected individuals release pro-inflammatory mediators, such as IL-1β and TNF-α, which contribute to chronic immune activation – a hallmark of HIV pathogenesis. Platelets also support the survival and replication of the virus by interacting with infected immune cells, contributing to the establishment of viral reservoirs. This interaction suggests that platelets play a significant role in the chronic inflammation seen in HIV and may represent a therapeutic target to reduce viral persistence and immune activation[44].

## Platelet-derived microparticles (PMPS) and conflicting findings regarding platelet-leukocyte aggregates

Although the current literature provides strong evidence regarding the complex functions of platelets in inflammation, coagulation, and immunity, various aspects necessitate deeper analysis and thorough evaluation. A notably significant element is the function of platelet-derived microparticles (PMPs), which have arisen as crucial agents of intercellular communication in both HIV and SCD. PMPs contain bioactive molecules such as cytokines, chemokines, RNA transcripts, and coagulation factors, and have the ability to influence immune and endothelial cell responses. Nonetheless, many research efforts focused on PMPs face constraints due to small sample sizes, differences in isolation methods, and insufficient standardization in quantification approaches. Such inconsistencies hinder the reproducibility of results and complicate comparisons across studies^[40,41]^. In the realm of HIV, heightened levels of PMPs have been linked to endothelial dysfunction, immune activation, and advancement of cardiovascular complications. However, some studies indicate no significant link between PMP concentration and viral load or immune status, which raises concerns regarding their diagnostic and prognostic reliability. Likewise, in SCD, elevated PMP levels are linked to the severity of the disease and the occurrence of vaso-occlusive crises; however, other research indicates that microparticles originating from red blood cells or monocytes might play a more significant role in advancing pathogenesis. This variation in results highlights the necessity for more standardized approaches and longitudinal research to more clearly identify the specific functions and biomarkers linked to PMPs in both conditions[42].

A different area of contradictory evidence centers on the function of platelet-leukocyte aggregates (PLAs), especially regarding their role in chronic inflammation and immune imbalance. Several studies emphasize the heightened creation of PLAs in both HIV and SCD, indicating their role as enhancers of pro-inflammatory signaling and vascular damage. These aggregates are recognized for promoting leukocyte activation, transmigration, and cytokine release, thus leading to endothelial dysfunction and tissue injury. Nonetheless, contradictory information exists concerning the stability and importance of these aggregates throughout different disease stages. Certain studies indicate that PLAs may be temporary and could represent acute-phase responses instead of ongoing immune activation, whereas other research argues that their sustained increase directly contributes to disease advancement[43]. Additionally, variations in assay sensitivity, flow cytometry gating approaches, and timing of sample collection might explain the inconsistent results observed in studies assessing PLAs. In HIV, for instance, ART-treated individuals can exhibit differing PLA levels based on the degree of immune reconstitution, complicating the establishment of a reliable link with clinical outcomes. In SCD, the development of PLAs seems to change with the state of the crisis, but there is a lack of longitudinal studies to evaluate their temporal dynamics. Many studies in this field also depend on observational or in vitro designs, restricting causal conclusions. Limited functional studies investigating the mechanistic pathways connecting PMPs and PLAs to disease pathology exist, especially concerning HIV-SCD coinfection^[44,45]^.

## Limitations of the review

Although this narrative review provides an extensive look at the complex role of platelets in HIV and SCD, it is crucial to recognize some inherent limitations. Primarily, the narrative aspect of this review could lead to selection bias, since the choice and focus of studies were determined by the authors’ judgment instead of a set and replicable systematic approach. In contrast to systematic reviews, narrative reviews tend to be more influenced by subjective judgment and might miss pertinent findings because they lack established inclusion/exclusion criteria. Additionally, the review fails to include a quantitative synthesis or meta-analysis and does not utilize a formal quality evaluation of the studies that were included. Consequently, although the article combines mechanistic insights and up-to-date clinical information, the lack of organized data aggregation restricts its capacity to make statistically backed conclusions or evaluate the comparative strength of the existing evidence.

Another major limitation is the lack of data specifically focused on platelet biology in relation to HIV-SCD coinfection. Although new evidence indicates overlapping and possibly synergistic processes of platelet activation and immune dysfunction in coinfected individuals, limited research has directly examined this relationship. This limitation impedes a more thorough examination of disease-specific variations or treatment implications in this subgroup. Moreover, the diversity of the existing literature – spanning in vitro experiments, animal studies, and small human groups – restricts the broad applicability of certain mechanistic findings. Additionally, differences in patient demographics, disease stages, antiretroviral therapy statuses, and definitions of platelet dysfunction among studies contribute to the complexity of interpretation. Ultimately, while the review aims to include the latest and pertinent literature, publication bias and the dominance of results from specific geographic areas or research teams may unintentionally distort the overall narrative. This is particularly relevant in low- and middle-income nations, where both HIV and SCD are most common, yet research productivity may be constrained.

## Conclusion

Platelets, once primarily viewed as players in hemostasis, are now recognized as pivotal modulators of the immune system. Their ability to interact with a variety of immune cells, release cytokines and chemokines, and influence both the innate and adaptive immune responses highlights their complex and multifaceted role in immune regulation. Through these mechanisms, platelets contribute to both the initiation and resolution of inflammation, maintaining immune homeostasis under normal conditions. However, when platelet activation becomes dysregulated, it can lead to the exacerbation of inflammatory diseases, autoimmune disorders, and infections. In conditions such as HIV, SCD, and other chronic inflammatory diseases, platelets contribute to immune activation, thrombosis, and tissue damage. This underscores the importance of understanding platelet function in these contexts to develop targeted therapeutic strategies. The dual role of platelets in promoting immune responses while also aiding in their resolution presents both a challenge and an opportunity for therapeutic interventions. By selectively modulating platelet activity, it may be possible to reduce harmful inflammation, improve disease outcomes, and enhance patient care.

## Data Availability

Not applicable as this a Narrative Review.

## Research registration unique identifying number (UIN)

Not applicable as this a Narrative Review.
